# Mediators of improved child diet quality following a health promotion intervention: the Melbourne InFANT Program

**DOI:** 10.1186/s12966-014-0137-5

**Published:** 2014-11-04

**Authors:** Alison C Spence, Karen J Campbell, David A Crawford, Sarah A McNaughton, Kylie D Hesketh

**Affiliations:** Centre for Physical Activity and Nutrition Research, School of Exercise and Nutrition Sciences, Deakin University, 221 Burwood Hwy, Burwood, Victoria 3125 Australia

**Keywords:** Mediators, Diet quality, Child, Health promotion intervention

## Abstract

**Background:**

Young children’s diets are currently suboptimal. Given that mothers have a critical influence on children’ diets, they are typically a target of interventions to improve early childhood nutrition. Understanding the maternal factors which mediate an intervention’s effect on young children’s diets is important, but has not been well investigated. This research aimed to test whether maternal feeding knowledge, maternal feeding practices, maternal self-efficacy, and maternal dietary intakes acted as mediators of the effect of an intervention to improve child diet quality.

**Methods:**

The Melbourne Infant Feeding Activity and Nutrition Trial (InFANT) Program was a cluster-randomized controlled trial, conducted from 2008–2010. This novel, low-dose, health promotion intervention was delivered quarterly over 15 months and involved educational activities, promotion of peer discussion, a DVD and written materials. Post-intervention, when children were approximately 18 months of age, child diets were assessed using multiple 24-hour recalls and a purpose-developed index of diet quality, the Obesity Protective Dietary Index. Maternal mediators were assessed using a combination of previously validated and purpose-deigned tools. Mediation analysis was conducted using the test of joint significance and difference of coefficients methods.

**Results:**

Across 62 parents’ groups in Melbourne, Australia, 542 parents were recruited. Post- intervention, higher maternal feeding knowledge and lower use of foods as rewards was found to mediate the direct intervention effect on child diet quality. While other aspects of maternal feeding practices, self-efficacy and dietary intakes did not act as mediators, they were associated with child diet quality.

**Conclusions:**

Mediation analysis of this novel health promotion intervention showed the importance of maternal feeding knowledge and use of foods as rewards in impacting child diet quality. The other maternal factors assessed were appropriate targets but further research on how to impact these in an intervention is important. This evidence of intervention efficacy and mediation provides important insights for planning future interventions.

**Trial registration:**

Current Controlled Trials ISRCTN81847050, registered 23 November 2007.

**Electronic supplementary material:**

The online version of this article (doi:10.1186/s12966-014-0137-5) contains supplementary material, which is available to authorized users.

## Background

Dietary intakes of young children are suboptimal [1-3]. Low intakes of fruits and vegetables and high intakes of energy-dense, nutrient-poor (non-core) foods in early childhood are likely contributing to high rates of overweight, obesity and chronic disease across the lifespan [4-6]. Interventions to improve these aspects of young children’s diets are therefore vital.

Health promotion interventions for young children are most likely to be effective if they involve parents [7,8], and target factors likely to be important influences on children’s diets. Maternal nutrition knowledge has shown associations with better child diet quality in preschoolers [9,10]. Child feeding practices which have shown associations with healthier diets of young children include lower parental pressure [11], lower parental control of feeding [12], higher parental covert and overt control [13], higher child control [14], and lower use of foods as rewards [14-16]. Maternal self-efficacy in child feeding has shown associations with higher children’s vegetable intakes [17], and lower child intake of non-core foods [17,18]. Furthermore, maternal role modeling of dietary intakes, reflected by maternal diet, is associated with young children’s diets, for example, for fruits and vegetables [12,16,19,20], and sweets and soft drinks [16]. When providing a child health promotion intervention to parents, it is therefore likely to be important to target these constructs. Improvements in these maternal constructs should promote improved child diets, however, these hypotheses have been infrequently assessed, particularly with parents of children under five years of age.

Mediation analysis facilitates the assessment of *how* an intervention effect was achieved, and an understanding of which aspects of an intervention were most effective or important and which may need improvement. In statistical terms, mediation analysis seeks to identify an intermediate variable in the causal sequence relating an independent variable to an outcome variable [21]. By testing the theories on which an intervention is based, mediation analysis can assist in the interpretation of trial results and can inform the development of more parsimonious future interventions [21]. The importance of mediation analyses to assess intervention and prevention trials has been previously highlighted [22-25], however, relatively few studies have undertaken such analyses of dietary interventions in children [26].

**Figure 1:**
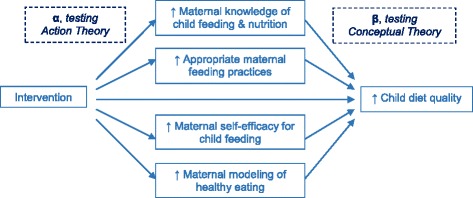
**Theoretical model showing proposed mediators of the intervention effect on child diet quality, informed by MacKinnon 2008** **[** 21 **]** **.**

The Melbourne Infant Feeding Activity and Nutrition Trial (InFANT) Program was a health promotion intervention which significantly improved diet quality of children at 18 months of age [27]. The intervention aimed to achieve this via improving mothers’ knowledge of child feeding and nutrition, child feeding practices, self-efficacy for promoting healthy eating and modeling. Therefore the hypothesis tested in this study is that these maternal correlates would act as mediators of the intervention effect on children’s diet quality, as outlined in Figure 1. This study aims to assess which components of maternal behavior were impacted by the intervention and acted as mediators of the intervention effect on child diet quality.

## Methods

The Melbourne InFANT Program was a cluster-randomized controlled trial to promote obesity-protective behaviors in the early childhood years, conducted from 2008–2010 [28,29]. The intervention targeted nutrition, physical activity and sedentary behavior, but only nutrition and feeding-related outcomes will be discussed in this paper. The study was conducted through the existing social setting of first-time parents’ groups, which are run within local government areas (LGAs) in Victoria, Australia, by Maternal and Child Health nurses (about two thirds of new mothers attend [30]). Fourteen randomly selected LGAs were recruited, then parents’ groups within those LGAs were randomly selected and approached. Groups with at least eight eligible families (English-speaking, first-time parents) who provided written, informed consent, were randomized to either the intervention or control arm. An independent statistician undertook all stages of randomization using a random number schedule, to ensure there were no biases in allocation. Mothers and fathers were both permitted to participate, but most attendees were mothers, who are the focus of this paper.

The intervention involved six interactive sessions over 15 months, from when children were approximately four months of age. Sessions included educational activities, a DVD, promotion of peer discussion, and written materials. The intervention had a major focus on improving children’s diet quality (specifically promoting fruits and vegetables and discouraging non-core foods), via improving maternal knowledge, feeding practices, self-efficacy and role modeling. Key principles of the intervention included offering anticipatory guidance [31], promoting division of responsibility in child feeding [32] and emphasizing parenting skills [28]. Participants in the control arm received quarterly newsletters on topics unrelated to the intervention, in addition to the usual care available to them in their local area from their Maternal and Child Health nurse. Ethics approval was granted by the Deakin University Ethics Committee and the Victorian Office for Children.

### Outcome measures

#### Demographic data collection

Mothers completed a self-administered questionnaire at baseline, when children were approximately four months of age. Data was collected regarding the child’s birth date and birth weight, as well as mothers’ education level, self-reported pre-pregnancy height and weight (utilized to calculate maternal BMI), and birth date (utilized to calculate maternal age at childbirth).

### Maternal mediators

Eighteen potential maternal mediators were assessed via self-completed questionnaire post-intervention. All individual items were grouped into summed scores or factors to provide more robust and meaningful outcomes for analyses, and to limit statistical issues associated with multiple comparisons. The 14 items purpose-designed for the current study are listed in Additional file 1. Test-retest reliability was assessed in a separate sample of 51 mothers with children aged approximately 18 months, for the amended and newly developed items/factors and those which hadn’t previously been validated in an Australian population this age. Test-retest questionnaires were administered two weeks apart, and intraclass correlations (ICC) between time points ranged from 0.65 to 0.86 (presented in Table 1).

**Table 1:** **Summary of measures assessing potential maternal mediators**

**Maternal mediators**	**Source**	**Cronbach’s α (InFANT)**	**ICC (reliability study)**
Knowledge of child feeding messages (12 items)	Purpose-designed	N/A (summed score)	0.73
Feeding practices	Child Feeding Practices Questionnaire [33]		
Use of pressure in feeding (4 items)		0.64	0.82
Use food as reward (2 items)^a^		0.69	0.66
Restriction (4 items)		0.65	0.71
Intentional modelling of healthy eating (4 items)		0.79	0.71
Encourage balance and variety (2 items)		0.67	0.65
Emotion regulation (3 items)		0.65	0.71
Child control (5 items)^b^		0.52	0.80
Covert control (5 items)	Ogden et al. [13]	0.79	0.86
Self-efficacy	Purpose-designed and previously utilized		
Confidence in promoting healthy foods (4 items)		0.68	0.70
Confidence in limiting unhealthy foods (3 items)		0.86	0.74
Confidence in providing healthy eating settings (2 items)		0.60	0.70
Maternal diet (7 components)	Anti-Cancer Council Victoria FFQ	N/A (summed frequencies)	N/A^c^

^a^One item dropped to improve Cronbach’s α from 0.60.

^b^Factor not used in analyses due to low Cronbach’s α, which was not improved by item removal.

^c^Previously validated in similar population group [35,36].

### Maternal knowledge of child feeding intervention messages

Maternal knowledge of child feeding intervention messages was assessed via a purpose-designed 12-item tool, as there were no previously validated tools available (see Table 1 for ICC and Additional file 1 for questions). Three experts involved in the intervention design constructed these items, specifically to assess maternal knowledge of the key intervention messages around child feeding and nutrition. Responses to each item were coded as correct (score 1) or incorrect (score 0), and scores were summed to provide a knowledge score out of 12. Scores were not normally distributed, therefore they were grouped into three categories for analysis based on the data distribution: ≤8; 9–10; and 11–12.

### Maternal feeding practices

Maternal feeding practices were assessed using six subscales from the Comprehensive Feeding Practices Questionnaire (CFPQ), which has been validated with children as young as 18 months [33]. Each item has a five-point response scale (score range 0–4), and subscale scores were calculated as recommended, by taking the mean score of the subscale items [33]. The subscales utilized in the current study were those which experts deemed relevant to children at 18 months of age, which showed adequate internal reliability (Cronbach’s α >0.60 [34]), and which demonstrated adequate test-retest reliability (ICC >0.60 [34]) (details presented in Table 1). These included use of pressure in feeding, use of foods as rewards (i.e. offering favorite food/sweets for good behavior), restriction, modeling, encouraging balance and variety, and use of food for emotion regulation. The child control subscale of the CFPQ was assessed but not included in analyses because reliability was low (Cronbach’s α =0.52). Scores were not normally distributed, and thus were grouped into three categories for analysis based on distinguishing theoretically meaningful categories: 0–1 (answers mostly indicating disagreement or low frequency); >1 to <3 (answers mostly representing neutral or sometimes); and 3–4 (answers mostly indicating agreement or high frequency). In addition to CFPQ subscales, items pertaining to a factor assessing covert control of child eating [13] were also included as part of the assessment of feeding practices (Cronbach’s α and ICC presented in Table 1). This factor was scored and categorized in the same way as the factors from the CFPQ.

### Maternal self-efficacy for healthy child feeding

Maternal self-efficacy for healthy child feeding was assessed using a combination of seven previously developed items [17], and two purpose-designed items (included in Additional file 1). These assessed confidence in promoting healthy child diets and undertaking healthy feeding practices. Responses on a four-point response scale ranged from ‘not at all confident’ (score of 0) to ‘extremely confident’ (score of 3). Factor analysis (using exploratory principal factor analysis with varimax rotation) revealed three factors with acceptable internal reliability and ICCs. These were labeled confidence in: promoting healthy foods (comprising four items); limiting unhealthy foods (three items); and providing healthy eating settings (two items). Mean scores were calculated for each self-efficacy factor, and as distributions were not normal, scores were grouped into three categories for analysis based on the data distribution and distinguishing theoretically meaningful categories: 0-<1.5 (answers mostly indicating ‘not at all/slightly confident’ or ‘strongly agree/agree’ with barriers); 1.5 to <2.5 (very confident); and 2.5-3 (extremely confident).

### Maternal diet (modeling)

Maternal diet, as a measure of modeling, was assessed using a food frequency questionnaire (FFQ) previously validated in a sample of Australian women [35,36]. This FFQ assesses usual frequency of intake of 98 food items over the preceding 12 months, using a 10-point scale ranging from ‘never’ to ‘three or more times per day’. Additionally, daily serves of specific foods and food groups are assessed. The focus of maternal dietary assessment was fruits, vegetables and non-core foods given these were the focus of the intervention. As this study aims to assess which components of maternal behavior may act as mediators, the food groups were assessed separately rather than as a combined index. It is feasible that a change in one aspect of maternal diet may impact on more than one aspect of child diet [37,38], and hence act as a mediator of child diet quality.

Daily serves of fruit and vegetables were assessed by two items (In the last 12 months, how many serves of fruit/vegetables did you usually eat per day?), with 8 and 9 point response scales respectively from none to 6/7 or more serves per day. Intakes were dichotomized for analysis, at two serves/day for fruit (based on meeting the Australian Dietary Guidelines recommendations for adults [39]), and at three serves/day for vegetables (dichotomized at the sample median due to the low number of people meeting the recommended five serves/day [39]). Additionally, fruit variety (out of 21) and vegetable variety (out of 31) scores were calculated by combining frequency data for individual items (sum of varieties consumed at least once per month). Due to non-normal distributions of the variety outcomes, scores were dichotomized at the sample mean for the purposes of analyses.

For assessment of maternal non-core foods, intakes of relevant items were converted to Daily Equivalent Frequencies (DEFs), as per instructions for use of the FFQ (for example, a response of 3–4 times per week was given a value of 0.5 DEF) [40]. Frequencies of intake were assessed by summing the DEFs of relevant questionnaire items, defined as: non-core drinks (regular soft drink, orange juice, other juices); non-core sweet snacks (cakes, sweet biscuits, ice cream, chocolate, other confectionary); and non-core savory snacks (non-wholemeal crackers, chips/crisps). The summed DEFs were then dichotomized for analysis to <1/day and ≥1/day for drinks and sweet snacks. Frequency of consuming savory snacks was lower, so these were dichotomized to <1/week and ≥1/week.

### Child dietary index

Parents completed 3 unscheduled telephone 24-hour recalls post-intervention. A purpose-designed food measurement booklet assisted parents with quantity estimation [27]. Recalls were conducted by trained, blinded research staff using the 5-pass method [3,41] and a purpose-designed database. Food item coding was also completed by trained, blinded staff, using the Australian Food Supplement and Nutrient Database (“AUSNUT2007”) [42], which contained foods relevant for young children, and was updated with infant-specific foods when necessary for this study. Completion and accuracy of coding of all interviews was checked by a dietitian. Participants were included in analyses if they completed 2 (n =26) or 3 (n =372) days of recalls, but outliers were excluded if mean energy intake was further than three standard deviations from the sample mean (n = 3), similarly to previous studies [27,43,44].

The assessment of such interventions using a dietary index allows relevant dietary targets to be considered together as a combined outcome measure [45]. Previously published diet quality indices appropriate for this population did not assess serves of non-core foods [46-53]. Therefore, an Obesity Protective Dietary Index (OPDI) was created to incorporate the key dietary targets of the Melbourne InFANT Program [27], and to reflect some key dietary components linked to obesity risk [6]. Average child daily intakes of fruits (grams), vegetables (grams) and non-core foods (kJ from both foods and beverages) were calculated. The fruit and vegetable groups included fresh, dried and tinned products (not juice), and contributions from mixed dishes, calculated using an approach similar to the disaggregation method to calculate MyPyramid Food Groups by the United States Department of Agriculture [54]. The non-core food group consisted primarily of foods and beverages likely to be eaten as snacks, including juice, soft drink, cordial, sweetened milks, sweet & savory biscuits, crisps, confectionary, cakes, pastries, buns and takeaway foods.

To calculate OPDI scores, all children’s fruit intakes were ranked, then divided into 11 quantiles, and allocated scores of 0–10 based on quantile ranking (with 10 representing the highest consumers). This method has been previously employed to assign dietary variety scores within the Healthy Eating Index [55]. This process was repeated for intakes of vegetables and non-core foods (scoring for non-core foods was reversed, with a score of 10 allocated to the lowest consumers). Scores for the three components were then summed to give a total ODPI score out of 30. Scores have previously been reported elsewhere, with a modest difference between children in the intervention arm (15.6 ± 5.9) compared to the control arm (14.5 ± 6.7) (p =0.01) [27]. Score validity was assessed by testing associations between OPDI scores and intakes of energy, fiber, and relevant nutrients (saturated fat, β-carotene, vitamin C and sodium). Pearson’s correlation coefficients were all in the hypothesized direction and ranged from −0.11 (sodium) to 0.55 (fiber) (adjusted for energy intake) [27].

### Statistical analysis

Analyses were conducted using Stata, version 11.1 software. Prior to conducting mediation analyses, factors were tested for multi-collinearity using bivariate correlations [56], and correlations between factors were confirmed not to be greater than 0.6 [56]. Covariates included in all analyses were maternal age at childbirth, maternal education level, child age (at date of first recall), child energy intake, and clustering (by first-time parents’ group), which is common in mediation analyses when recruitment is conducted at a group level [57,58]. Participants who provided complete data for all variables in each model were included in analysis, and intention-to-treat principles for analysis of completers were employed.

Two tests of mediation were employed in this study. The joint significance test [21,59-61] has previously been utilized for assessing mediators with non-continuous data [59], and does not specify any requirements or assumptions for the distribution of variables. Additionally this test minimizes Type 1 error and maximizes statistical power [60]. The two steps in testing joint significance are: 1) assessment of the association between the independent and mediator variables (α pathway), and 2) assessment of the association between the mediator and the outcome variables (β pathway) [60,61], as shown in Figure 1. For step 1, there must be a significant association between the independent variable (in this case, treatment arm, i.e. intervention or control), and the mediator (in this case, each of the maternal outcomes), represented by α in Figure 1. Data for all potential mediator variables was categorized for analysis (as described above, based on the data distributions and/or theoretically meaningful categories), thus the α-pathway was tested by ordered logistic regression or binary logistic regression. For step 2, there must be a significant association between the mediator and the outcome (in this case, child OPDI score) when controlling for the independent variable. This is represented by β in Figure 1, and was tested using linear regression, as OPDI scores were normally distributed. Joint significance of both pathways indicates mediation.

As the test of joint significance does not provide an estimate of the effect size or confidence intervals of the mediated effect [60], the difference of coefficients test was then conducted for those variables identified as mediators by the test of joint significance, to address these limitations [21,60]. The direct effect of the intervention on the outcome (c) was firstly assessed. For each mediator, the effect of the intervention on the outcome when controlling for the mediator (c’) was also calculated. To determine the effect of the mediator (i.e. the indirect effect), the difference between these coefficients (c-c’) was then calculated with bias-corrected bootstrap analyses (2000 replications) [21]. Significance for the bias-corrected indirect effect was assessed. The percentage of the total intervention effect explained by the mediator was also calculated ((c-c’)/c*100). All calculations of c and c’ were conducted with only those participants with complete data for all mediators.

## Results

The Melbourne InFANT Program recruited 542 families from 62 parents’ groups, with 528 of those being eligible first-time parents (intervention n =262, control n =266). The participant flowchart and sample details have been provided elsewhere [29], and characteristics of the sample are presented in Table 2. There were no differences in demographic characteristics between intervention and control arms at baseline [29].

**Table 2:** **Characteristics of participants in intervention and control arms at baseline**

	**Total sample n (528)** ^**a**^	**Intervention n (262)** ^**a**^	**Control n (266)** ^**a**^
Male children	53%	52%	54%
Child age at baseline (months) (mean ± SD)	3.6 ± 1.0	3.7 ± 1.1	3.6 ± 1.0
Child birth weight (grams) (mean ± SD)	3382 ± 593	3393 ± 547	3371 ± 636
Maternal age at childbirth (years) (mean ± SD)	31.9 ± 4.3	32.1 ± 4.2	31.7 ± 4.5
Maternal pre-pregnancy BMI (kg/m^2^) (mean (IQR))	23.1 (20.6 – 26.7)	23.4 (20.6 – 27.0)	23.0 (20.6 – 26.6)
Maternal education at baseline:			
High school education or lower	21%	22%	20%
Diploma or trade certificate	25%	26%	23%
Tertiary qualification	55%	52%	57%

^a^Number of participants recruited is presented. Not all participants provided complete data at baseline, thus n = 502-522 for the total sample for the variables presented.

Post-intervention, children were 18.0 ± 1.5 months of age (at first 24-hour recall). Of the 480 participants (91% of baseline sample) who completed the trial, complete data for child diet recalls and at least one maternal mediator was provided by 375 mothers (71% of baseline sample). Those participants who provided complete data were more likely to have a higher education level than those who discontinued the study or provided insufficient data (data not shown). However, there were no differences in completion rates by pre-pregnancy BMI or maternal age at childbirth.

Significant differences between intervention and control arms (in the expected direction) were seen for four of the mediators (α pathways): maternal knowledge of child feeding intervention messages and intentional modeling of healthy eating (higher in the intervention arm), and use of foods as rewards and use of pressure in feeding (lower in the intervention arm), as shown in Table 3. One further item showed a trend towards significance: maternal confidence in limiting unhealthy foods.

**Table 3:** **Mediation of child Obesity Protective Dietary Index scores post-intervention** ^**a**^

**Maternal mediators**	**n**	**α**	**(95% CI)**	**β**	**(95% CI)**	**c’**	**Indirect effect (c-c’)**	**P-value for bias-corrected indirect effect**	**% effect explained**
Knowledge of child feeding messages	362	0.99	(0.59, 1.38)**	3.12	(0.90, 5.35)**	1.00	0.50	0.003	33%
Feeding practices									
Use of pressure in feeding	371	−0.51	(−1.00, −0.02)**	−2.16	(−4.35, 0.03)*				
Use food as reward	374	−0.77	(−1.47, −0.07)**	−3.69	(−6.10, −1.27)**	1.17*	0.33	0.016	22%
Restriction	361	0.17	(−0.23, 0.56)	−0.99	(−3.19, 1.21)				
Intentional modelling of healthy eating	375	0.51	(0.01, 1.00)**	8.00	(2.69, 13.3)**	1.31**	0.19	0.082	12%
Encourage balance and variety	375	0.39	(−2.08, 2.86)	2.31	(−2.48, 7.10)				
Emotion regulation	362	0.14	(−0.22, 0.50)	−5.58	(−6.83, −4.33)**				
Covert control	362	0.15	(−0.25, 0.56)	2.85	(0.75, 4.95)**				
Self-efficacy									
Confidence in promoting healthy foods	367	0.24	(−0.22, 0.69)	6.33	(2.16, 10.49)**				
Confidence in limiting unhealthy foods	367	0.39	(−0.00, 0.78)*	3.00	(1.02, 4.97)**				
Confidence in providing healthy eating settings	366	0.00	(−0.38, 0.38)	1.63	(−0.67, 3.92)				
Maternal diet									
Fruit intake	359	0.29	(−0.33, 0.90)	1.33	(−1.19, 3.86)				
Vegetable intake	359	0.11	(−0.32, 0.54)	1.34	(0.06, 2.62)**				
Fruit variety	358	−0.10	(−0.46, 0.44)	0.95	(−0.29, 2.18)				
Vegetable variety (with potato)	357	0.04	(−0.42, 0.50)	0.62	(−0.66, 1.89)				
Non-core drink intake	358	−0.08	(−0.58, 0.43)	−1.34	(−2.71, 0.03)*				
Non-core sweets intake	357	−0.16	(−.054, 0.22)	−1.57	(−2.77, −0.37)**				
Non-core savory foods intake	357	0.21	(−0.24, 0.66)	−1.35	(−2.69, 0.00)*				

^a^All analyses controlled for daily mean energy intake, child age at the first recall, maternal education level, maternal age at childbirth and clustering by parent group.

*p <0.10, **p <0.05.

The relationship between the maternal mediators and child OPDI score (β pathways) was significant for nine of the 18 pathways, and three further pathways showed trends towards significance. The β values in Table 3 indicate the difference in ODPI score between the highest and lowest category of the mediator. All trends and associations were in the hypothesized direction.

Joint significance was therefore demonstrated for three mediators, as shown in Table 3. The difference of coefficients test was then conducted for these three mediators. The direct intervention effect (c) for the sample with complete data for all three mediators was 1.50 (p =0.01, n =357). The indirect effects (c-c’) for each mediator are shown in Table 3. Higher maternal knowledge of child feeding intervention messages and lower maternal use of food as a reward in the intervention compared to control arm mediated the intervention effect on child OPDI score, explaining 33% and 22% of the intervention effect respectively. Additionally, use of pressure in feeding, intentional modelling, and self-efficacy for limiting unhealthy foods could be considered to show trends towards mediation of the intervention effect, with trends towards significant α and β pathways.

## Discussion

These analyses are novel and valuable because they inform the targets and content of future interventions by highlighting maternal domains likely to be most important to achieving an impact on young children’s diets. The results indicate that higher maternal knowledge of child feeding and nutrition may contribute to small improvements in child diet quality, even with minimal concurrent improvements in maternal behaviors and self-efficacy. The finding of knowledge as a mediator of the intervention effect is consistent with other health promotion studies in older children and adults [62-64]. An important distinction and strength of this study, however, was that knowledge was measured by a set of 12 items, rather than a single item. Measurement using a factor comprised of multiple items is likely to be more robust than individual item analysis. The cumulative results of these studies highlight the value and importance of improvements in nutrition knowledge, and suggest that maternal knowledge *can* directly influence children’s diets, but that the effect of knowledge alone may be quite small. In this study, higher knowledge in the intervention arm explained 33% of the modest intervention effect on child diet quality.

The finding that lower use of food as a reward mediated the intervention effect is novel and informative. While one other study reports that improvements in parental feeding strategies mediated the effects of a nutrition promotion intervention on young children’s non-core foods intakes, it assessed a composite of feeding practices as the mediator rather than distinguishing between practices [18]. Two further studies in a similar age group have also reported intervention effects on parental use of food as a reward [65,66], though those did not assess mediation. Considered together, these studies highlight the benefits of including parental feeding practices as targets of an intervention, and a need for further investigation of whether and why some feeding practices may be more amenable to interventions than others.

It is not known why reduced use of food as a reward acted as a mediator while other feeding practices did not. It was not emphasized more strongly in the intervention, and it did not show more need for improvement than other factors. Qualitative assessments and process evaluation of the intervention have also not identified any reason for this difference (findings not yet published). It is possible that this practice is particularly amenable to change. Following the Melbourne InFANT Program, in general it was the less healthy practices, or those not consistent with feeding recommendations, which differed between trial arms. Intervention participants used less food as a reward and less pressure, and trended towards being more confident to limit unhealthy foods, with minimal impact on improving healthy behaviors. Intervention effects on child TV and non-core sweet snacks, and mothers’ unhealthy dietary patterns, have previously been reported, but with no impact on child or maternal physical activity or fruit and vegetable intakes [29,67]. It is possible that participants found it easier to reduce their unhealthy behaviors than to increase their healthy behaviors.

Important findings from these mediation analyses were the significant associations between many of the potential mediators and child diet quality (as shown by significant β pathways). Such associations support the conceptual theory behind the choice of maternal targets of the Melbourne InFANT Program, as maternal factors were related to child diet quality in the expected direction, even if the maternal factors were not influenced by the intervention. These findings highlight that these are worthy intervention targets for future programs because they are likely to influence child diets. However, the action theory and strategies used to influence these maternal mediators may benefit from refinement in future interventions. For example, many aspects of maternal diet were associated with child diet, so if intervention impact on maternal diet could be improved, and this act as a mediator, the effect on child diet may be greater. These findings are similar to those of Fletcher et al., who found that parental self-efficacy for providing healthy foods was associated with young children’s intake of non-core foods, but was not improved by a nutrition promotion intervention [18].

Only one previous study is known to have assessed parental mediators of young children’s diets following a nutrition promotion intervention [18]. That study offered a one month telephone-based intervention for parents with children aged 3–5 years. It found that child access to non-core foods and child feeding strategies mediated reduced child non-core food intake at 2 months (assessed by FFQ), but there was no intervention effect at 6 months. The current study therefore extends this small evidence base by reporting a wider selection of parental mediators, assessing child diet quality as the primary outcome, evaluating a 15 month group-based intervention, and focusing on parents of children under two years. Other studies which have assessed mediation of child nutrition promotion interventions have primarily involved older children, and assessed children’s own knowledge, behaviors and beliefs as potential mediators [57,58,62,68-70]. While three of those papers did report parental diet and home fruit and vegetable availability [62,68,70], they were not found to be significant mediators of the intervention effect on child diets, likely due to the school-based nature of the studies and consequent minimal parental involvement.

A limitation of this study was the modest sample size, as this can limit the analytic techniques appropriate for mediation, and make it difficult to detect statistically significant mediation effects, particularly if the effect size is also small [71]. Additionally, those participants who provided adequate data for these analyses were more likely to have a higher education level than those who discontinued the study or provided insufficient data, which limits generalizability. It is also acknowledged that this single time-point data does not account for possible bi-directional relationships between maternal mediators and child dietary patterns [72]. However, longitudinal assessment was not applicable in this study, given that child feeding practices would not yet have been established at baseline when children were aged three months.

The unacceptable internal reliability of the child control subscale (Cronbach’s α 0.52) meant that this key aspect promoted within the intervention was not assessed as a mediator. In comparison, across the three validation studies in the United States, Cronbach’s α of this factor was variable: 0.49, 0.69 and 0.70 [33]. According to the principles of division of feeding responsibility [32], parents letting their child choose their foods from those served could be considered appropriate, and parents preparing alternatives if their child disliked what was served could be considered inappropriate. However, items assessing these behaviors load in the same direction on the factor, suggesting the factor may measure greater *allowance* of child control, not necessarily more *appropriate* child control. Further refinement and validation of tools to measure parental feeding practices in young children is required in future.

Strengths of this study include the high quality dietary assessment, modelled on the United States Department of Agriculture 5-pass system [73]. This method is used in national nutrition surveys [73,74], and was strengthened in this study by multiple days of recalls [75]. Additionally, use of a diet quality index is a novel strength of this study, allowing assessment of relevant dietary outcomes in combination, which is important for interventions which target multiple dietary behaviors [76,77]. Furthermore, the assessment of a variety of potential mediators, and the use of factors and scores rather than individual questionnaire items as mediators, distinguishes this study. This is also the first study known to have assessed maternal mediators of a health promotion intervention aimed at improving the diet of children under two years of age.

## Conclusions

Higher maternal knowledge of child feeding intervention messages, and lower maternal use of food as a reward in the intervention arm, mediated the effect of the Melbourne InFANT Program on child diet quality. This highlights the importance of these elements in public health nutrition interventions for parents. A number of other potential maternal mediators were shown to be appropriate intervention targets, but require further research to consider how they may be more effectively impacted in future interventions to promote behavior change.

## Additional file

Additional file 1:
**Questions included in the previously unpublished factors assessing maternal mediators.**

## Abbreviations

InFANT: Melbourne Infant Feeding Activity and Nutrition Trial
LGAs: Local Government Areas
ICC: Intraclass Correlations
CFPQ: Comprehensive Feeding Practices Questionnaire
FFQ: Food Frequency Questionnaire
DEFs: Daily Equivalent Frequencies
OPDI: Obesity Protective Dietary Index
