# Supplementary material for: Mediators of improved child diet quality following a health promotion intervention: the Melbourne InFANT Program
Source: Int J Behav Nutr Phys Act. 2014 Nov 4;11:137. doi: 10.1186/s12966-014-0137-5 (PMC4230360; doi:10.1186/s12966-014-0137-5)
Supplement: Additional file 1: — Questions included in the previously unpublished factors assessing maternal mediators. [file 12966_2014_137_MOESM1_ESM.pdf]

Additional file 1

Questions included in the previously unpublished factors assessing maternal mediators

Factor	Items included
Knowledge [a]	It will be important that my child learns to enjoy a wide range of fruits and vegetables
	The foods I make available to my child will affect what foods s/he comes to like
	Children are more likely to enjoy a food if they see their parents eating it
	If I keep on offering foods my child hasn't previously enjoyed s/he is likely to come to enjoy them
	A good way to get my child to eat healthy foods will be to offer a food treat as a reward (for example, offering dessert if s/he eats all their vegetables)
	The only drinks children need are milk and water
	Parents should include fruit or vegetables in all children's meals and snacks
	TV should be turned off when children are eating meals
	Parents should not make a fuss if their child doesn't eat their meal
	To get children to try foods they haven't enjoyed in the past, it' s good to offer them with favourite foods
	Parents should (not) offer other foods if their child doesn't eat their meal
	Parents should (not) encourage children to finish everything on their plate
Confidence for promoting healthy foods [b]	Get my child to eat a wide range of foods [c]
	Get my child to eat enough vegetables (this does not include potato or potato chips) [c]
	Get my child to eat enough fruit (this does not include fruit juice) [c]
	Get my child to drink plain water (with no flavours or juice added) [c]
Confidence for limiting unhealthy foods [b]	Say 'no' to my child's demands/fussing for potato chips/twisties/cheezels and similar foods [c]
	Say 'no' to my child's demands/fussing for sweet snacks, confectionary, lollies and/or ice-cream [c]
	Say 'no' to my child's demands/fussing for soft-drinks, fruit juice, cordials and other sweetened drinks [c]
Confidence for providing healthy eating settings [b]	Talk with other people who care for my child about what to feed him/her
	Eat meals with my child on most days

[a] All items assessing knowledge utilised a 4pt response scale (Strongly disagree – Strongly agree), dichotomised to correct or incorrect.

[b] All items assessing confidence utilised a 4pt response scale (Not at all confident – Extremely confident), starting with the question: "*How confident are you that you will be able to do the following things with your child over the next year?".*

[c] Seven of the nine items assessing confidence were from a previous study: Campbell K, Hesketh K, Silverii A, Abbott G. **Maternal self-efficacy regarding children's eating and sedentary behaviours in the early years: associations with children's food intake and sedentary behaviours**. *Int J Pediatr Obes*. 2010, 5:501-8.
